# Herbivore-induced volatile emission from old-growth black poplar trees under field conditions

**DOI:** 10.1038/s41598-019-43931-y

**Published:** 2019-05-22

**Authors:** Andrea Clavijo McCormick, Sandra Irmisch, G. Andreas Boeckler, Jonathan Gershenzon, Tobias G. Köllner, Sybille B. Unsicker

**Affiliations:** 1 Max Planck Institute for Chemical Ecology, Department of Biochemistry, Hans-Knöll-Straße 8, 07745 Jena, Germany; 2 Present Address: Massey University, College of Sciences, Tennent Drive, 4410 Palmerston North, New Zealand; 3 Present Address: Michael Smith Laboratories, University of British Columbia, 2185 East Mall, Vancouver, V6T 1Z4 BC Canada

**Keywords:** Food webs, Plant ecology

## Abstract

Herbivory is well known to trigger increased emission of volatile organic compounds (VOCs) from plants, but we know little about the responses of mature trees. We measured the volatiles emitted by leaves of old-growth black poplar (*Populus nigra*) trees after experimental damage by gypsy moth (*Lymantria dispar*) caterpillars in a floodplain forest, and studied the effect of herbivory on the transcript abundance of two genes involved in the biosynthesis of VOCs, and the accumulation of defence phytohormones. Herbivory significantly increased volatile emission from the experimentally damaged foliage, but not from adjacent undamaged leaves in the damaged branches (i.e., no systemic response). Methylbutyraldoximes, 4,8-dimethyl-1,3,7-nonatriene (DMNT), (*Z*)-3-hexenol and (*E*)-β-ocimene, amongst other compounds, were found to be important in distinguishing the blend of herbivore-damaged vs. undamaged leaves. Herbivory also increased expression of *PnTPS3* (described here for the first time) and *PnCYP79D6-v4* genes at the damaged sites, these genes encode for an (*E*)-β-ocimene synthase and a P450 enzyme involved in aldoxime formation, respectively, demonstrating *de novo* biosynthesis of the volatiles produced. Herbivore-damaged leaves had significantly higher levels of jasmonic acid and its conjugate (−)-jasmonic acid-isoleucine. This study shows that mature trees in the field have a robust response to herbivory, producing induced volatiles at the damaged sites even after previous natural herbivory and under changing environmental conditions, however, further studies are needed to establish whether the observed absence of systemic responses is typical of mature poplar trees or if specific conditions are required for their induction.

## Introduction

In nature, plants are under constant threat of herbivore attack and have developed a plethora of defence strategies. Plant defences can be either structural (such as thorns or waxes)^1,2^ or chemical (such as alkaloids or mustard oils)^3,4^, direct (having a direct effect on the herbivore like toxins or deterrents)^4,5^ or indirect (affecting the herbivore by attracting its natural enemies, e.g., extra floral nectaries or volatiles)^6,7^; constitutive (being permanently present)^1,2^ or induced (being triggered by herbivore attack)^6,8^. Herbivore-induced plant volatiles (HIPVs) are chemical defences triggered upon herbivore damage, having both direct and indirect effects on the herbivores causing them^5–7^.

HIPV biosynthesis and release are regulated by phytohormones, such as jasmonic acid (JA) and salicylic acid (SA), which initiate signalling cascades controlling the expression of multiple defence genes including those responsible for volatile production^6,9^. Phloem feeders typically activate SA-dependent responses, while chewing insects activate JA-dependent responses, although other phytohormones like ethylene and abscisic acid may play a role and crosstalk between pathways can occur^10,11^. HIPVs are chemically diverse, involving products of different biochemical pathways including saturated and unsaturated six-carbon aldehydes, alcohols and their esters (also known as green leaf volatiles - GLVs); terpenes (including monoterpenes, homoterpenes and sesquiterpenes), aromatics, and sulphur- or nitrogen-containing compounds among others^9,12^. The induced compounds are mainly emitted locally from damaged plant tissues, but can also be released systemically from undamaged plant parts both above-^13,14^ and below-ground^15^. Previous publications suggest that systemic volatiles amplify damage signals serving as long-distance cues for enemies of herbivorous insects (parasitoids and predators) and warning undamaged plant parts or neighbouring plants about potential herbivory^15–19^.

Our knowledge of plant volatiles, their regulation, and their ecological roles has increased vastly during the last two decades. However, for practical reasons the majority of the published research has been carried out with annual herbaceous plants under laboratory or greenhouse conditions. These studies do not reflect the diversity of life forms occurring in natural plant communities^20,21^. Moreover, biotic and abiotic factors can have a profound impact on plant volatile emissions^22–24^, highlighting the need for more studies to be conducted in the field or under field-like conditions.

Like other plants, trees release numerous volatiles after herbivore attack^25–29^, but are rarely studied in the field (but see^30–32^) due to their large size and longevity, which pose challenges for volatile collection. Poplar trees (*Populus spp*.) have become important model organisms in plant biology thanks to their rapid growth, prolific reproduction, ease of cloning, and the availability of the sequenced genome of *P. trichocarpa*, amongst other traits^33–35^. Therefore, they are ideal models to explore herbivore-induced volatile emission and its regulation in trees.

Previous studies have investigated the effect of herbivory on the volatile emissions of young poplar trees in the lab^36–38^, and characterised some of the genes and enzymes responsible for the biosynthesis of induced terpenes ^36,38,39^ and nitrogen-containing compounds^40,41^. However, it is not clear if the observed patterns reflect the behaviour of mature trees under natural conditions. Therefore, this research aimed to investigate herbivore-induced volatile emission and its regulation in mature poplar trees under field conditions. To fulfil this aim, we characterized the local and systemic volatile emission from old growth trees (>60 years old) after experimental herbivory by *Lymantria dispar*, measured the transcript abundance of two genes involved in volatile biosynthesis, and determined the effects of herbivory on phytohormone levels (SA, JA and its isoleucine conjugates JA-Ile-(−) and JA-Ile-(+)).

## Results

### Experimental vs. natural herbivory

**Figure 1:**
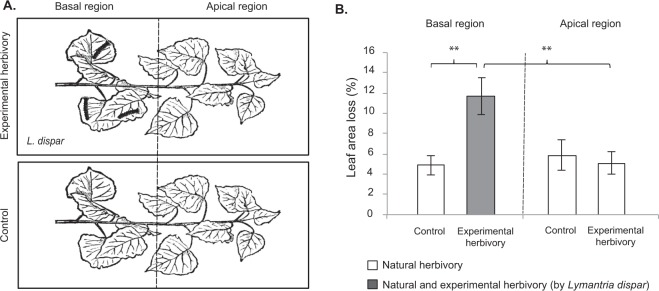
(**A**) Experimental setup showing the position of the *Lymantria dispar* caterpillars on the experimental herbivory treatment and the leaf sets sampled (apical and basal). Control samples for both positions (basal and apical) were taken from branches without experimental herbivory. (**B**) Average percentage of leaf area loss due to natural and experimental herbivory for the four leaf sets. Means ± SEM, n = 9. Asterisks (**) indicate significant differences between treatments with a probability value (p-value) below 0.01 after a paired T-test.

Experimental herbivory was carried out by confining *L. dispar* caterpillars on the basal region of a branch (Fig. 1A). This was done, to replicate an earlier experiment conducted with *P. nigra* saplings under controlled conditions where herbivore-damage was also applied in the basal leaves^36^. Four leaf sets were sampled: the apical and basal areas of experimentally damaged branches and the apical and basal areas of control branches without experimental herbivory (Fig. 1A).

After removing the *L. dispar* caterpillars, we photographed all the leaf pools and calculated the percentage of leaf area loss due to previous natural herbivory and experimental herbivory. Our results show that old-growth trees at the time of the experiment had already been subject to a considerable amount of previous herbivore damage (around 5% leaf area loss). The experimental herbivory caused approximately 7% of additional leaf area loss (12% total) and was significantly higher than the natural herbivory in the basal leaf pool of the control branches (Paired T-test; t = 4.957, d.f. = 1, p = 0.001) and the apical leaf pool of the same branch (Paired T-test; t = 3.487, d.f. = 1, p = 0.008) (Fig. 1B).

### Experimental herbivory induced an increase in volatile emission from the damaged sites

**Figure 2:**
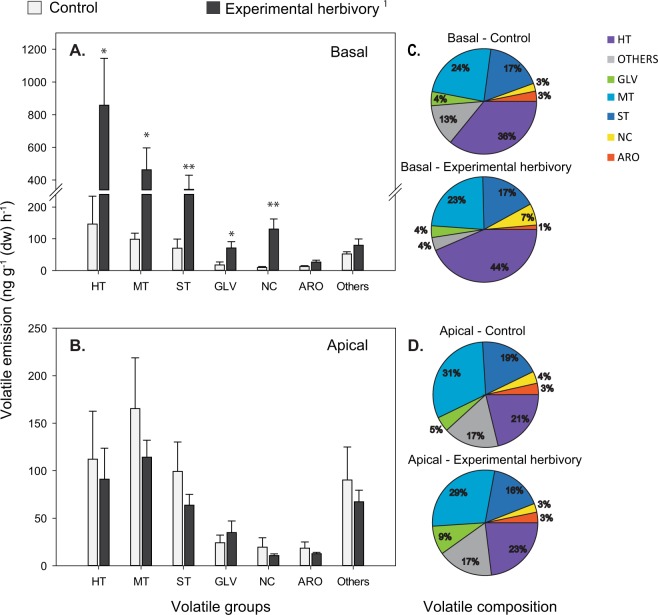
Emission of major groups of volatiles from basal and apical leaves of old-growth *Populus nigra* branches with and without experimental herbivory by *Lymantria dispar* (**A**,**B**). The pie charts (**C**,**D**) illustrate the relative proportion of the major groups of volatiles with respect to the full *P. nigra* odour blend. ^1^Experimental herbivory was only applied in the basal portion of the branch. Homoterpenes (HT), monoterpenes (MT), sesquiterpenes (ST), green leaf volatiles (GLV), nitrogenous compounds (NC), and aromatics (ARO). Means ± SEM. Asterisks indicate significant differences with probability values (p-values) below 0.05 (*) and 0.01 (**) after a Paired T-test. Statistical values are given in Table 1.

Over 50 volatile organic compounds were detected in the headspace of old-growth *P. nigra* trees (Table S1 Supporting Online Information). The majority of compounds identified belonged to the following chemical groups: homoterpenes (HT), monoterpenes (MT), sesquiterpenes (ST), green leaf volatiles (GLV), nitrogen-containing compounds (NC), and aromatics (ARO) (Fig. 2).

Experimental herbivory with *L. dispar* caterpillars feeding for 40 h *prior* to the 4 h volatile collection caused a significant increase in the emission of most groups of volatiles (HT, MT, ST, GLV, and NC) from experimentally damaged foliage (basal) in comparison to the foliage located in the same position in control branches (Fig. 2A, Table 1). However, there was no difference between the volatile emissions from the apical portions from *L. dispar*-damaged and control branches (Fig. 2B, Table 1).

**Table 1:** Results of a paired T-test, comparing the basal and apical areas of experimental herbivory vs. control branches.

*Chemical category*	Experimental herbivory vs. Control			
	Basal (n = 9)		Apical (n = 8)^a^	
	t^b^	p^c^	t^b^	p^c^
Monoterpenes	2.318	**0.049**	−0.601	0.566
Sesquiterpenes	3.786	**0.005**	−0.839	0.429
Homoterpenes	2.891	**0.020**	−0.527	0.614
Green leaf volatiles^d^	2.495	**0.030**	0.743	0.482^e^
Nitrogen-containing compounds	3.804	**0.005** ^**d**^	−0.804	0.448
Aromatics	2.220	0.057	−0.618	0.556
Other	1.360	0.209	−0.403	0.699

Highlighted values are statistically signficant at a 95% confidence interval (p < 0.05).

^a^Only eight pairs available due to the loss of one apical-control sample.

^b^t = T-statistic.

^c^p = probability value.

^d^Saturated and unsaturated six-carbon aldehydes, alcohols and their esters.

^e^Data were transformed using their natural logarithm (LN) to meet normality.

### Specific volatile compounds are indicative of local herbivore damage

To further explore the differences between the volatile emission of *L. dispar*-damaged and control leaves, we used a random forest analysis to compare the emissions of the basal leaf pools for both treatments. The analysis suggested 2- and 3-methylbutyraldoximes, α-copaene, (*E*)-4,8-dimethyl-1,3,7-nonatriene ((*E*)-DMNT), and eugenol amongst the key compounds being responsible for distinguishing between the two blends, having mean decrease in accuracy values above 75 (Table 2). These compounds were emitted in higher amounts in the experimental herbivory treatment and just present in minor amounts in the control treatment (except (*E*)-DMNT, which was also abundant in the control samples) (Table 2).

**Table 2:** Ranking of the 15 volatiles emitted from the basal portions of *P. nigra* branches that best distinguish the blends of the control and experimental herbivory treatments after a Random Forest Analysis, and their emission rates (nanograms per gram of dry weight per hour).

Rank	Compound	MDA^a^	Experimental Herbivory		Control	
			Emission	±SEM^b^	Emission	±SEM^b^
1	(*Z*)-2-Methylbutyraldoxime	82.156	14.206	0.249	0.497	0.249
2	(*E*)-2-+3-Methylbutyraldoxime	80.192	57.315	1.566	4.366	1.566
3	α-Copaene	82.156	6.494	0.588	2.039	0.588
4	(*E*)-DMNT^c^	77.337	774.039	86.664	145.140	86.664
5	Eugenol	78.975	4.780	0.121	0.233	0.121
6	Unidentified ST2^d^	50.38	18.274	1.651	3.278	1.651
7	(*Z*)-DMNT	50.055	16.891	1.204	2.792	1.204
8	(*Z*)-3-Hexenol	48.321	23.459	0.894	2.050	0.894
9	(Z)-3-Methylbutyraldoxime	46.89	11.511	0.497	1.783	0.497
10	(*E*)-β-Caryophyllene	46.004	41.295	4.267	13.692	4.267
11	(*E*)-β-Ocimene	45.747	22.067	2.174	6.495	2.174
12	(*Z*)-3-Hexenyl isovalerate	42.267	12.237	1.492	3.084	1.492
13	β-Cubebene	41.625	8.148	0.756	1.868	0.756
14	α-Humulene	35.326	12.099	0.868	3.057	0.868
15	α-Cubebene	31.671	3.105	0.314	0.703	0.314
	OOB^e^ error rate	11.11%				

^a^MDA = Mean decrease in accuracy.

^b^SEM = Standard error of the mean.

^c^DMNT = 4,8-dimethyl-1,3,7-nonatriene.

^d^ST = sesquiterpene.

^e^OOB = Out of bag error.

### Experimentally damaged foliage had higher transcript abundances of two genes involved in volatile biosynthesis

To test whether the emitted *P. nigra* volatiles were synthesized *de novo*, we measured the transcript levels of two genes involved in volatile biosynthesis: *PnCYP79D6-v4*, which encodes the methylbutyraldoxime-producing cytochrome P450^39^; and *PnTPS3*, which codes for the (*E*)-β-ocimene synthase. We selected these genes based on the results from previous studies showing that the compounds they encode: *i*) were induced by herbivory in poplar saplings, and *ii*) caused physiological and behavioural responses in the herbivore *L. dispar* and its parasitoid *Glyptapanteles liparidis*, suggesting that they are involved in direct and indirect tree defence^36,40^.

**Figure 3:**
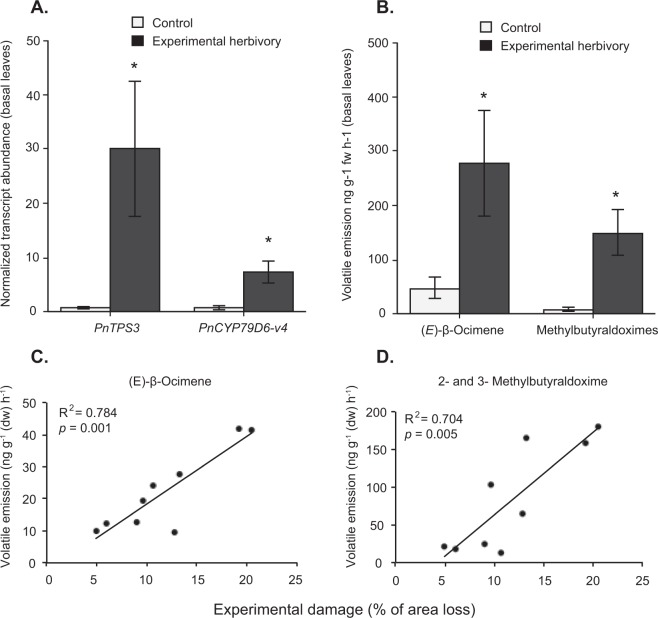
Transcript abundance of a terpene synthase (*PnTPS3*) and a cytochrome P450 enzyme (*PnCYP796-v4*) in the basal regions of control and experimental herbivory branches of six trees, and the corresponding emission rates of the reaction products, (*E*)-β-ocimene and 2- and 3-methylbutyraldoxime (n = 6) (**B**) Correlation between herbivore damage in the experimental herbivory treatment and (*E*)-β-ocimene as well as methylbutyraldoxime emission (**C**,**D**; n = 9). (**A**,**B**) Show mean values ± SEM. Asterisks (*) depict significant differences with a probability value (p-value) below 0.05 after a paired T-test.

The role of the *PnTPS3* product as an (*E*)-β-ocimene synthase was confirmed by cloning and heterologous expression of the *PtTPS6* orthologue from *P. trichocarpa*^40^ (Figure S1). The gene was named *PnTPS3* according to the terpene synthase nomenclature in *P. nigra*^36^. The transcript abundance of *PnTPS3* and *PnCYP79D6-v4* was assessed using qRT-PCR for the basal leaf pools only, in the experimental herbivory and control treatments. *PnTPS3* and *PnCYP79D6-v4* expression showed a 30-fold (paired T-test; t = 7.231, d.f. = 5, p < 0.001, data were ln-transformed) and 7-fold (paired T-test; t = 3.44, d.f. = 5, p = 0.018, data were ln-transformed) higher transcript abundance, respectively, in herbivore-damaged leaves compared to control leaves (Fig. 3A). This is in accordance with volatile emission of the enzyme products (*E*)-β-ocimene (paired T-test; t = 3.73, d.f. = 5, p = 0.013) and the methylbutyraldoxime isomers (T-test, t = 3.73, d.f. = 5, p = 0.013) in the corresponding leaf pools (Fig. 3B). The emission of these compounds was also positively correlated with the amount of leaf damage after a Pearson correlation test (Fig. 3C,D, Table S2).

### Experimental herbivory caused a significant increase in jasmonic acid levels at the damaged sites

**Figure 4:**
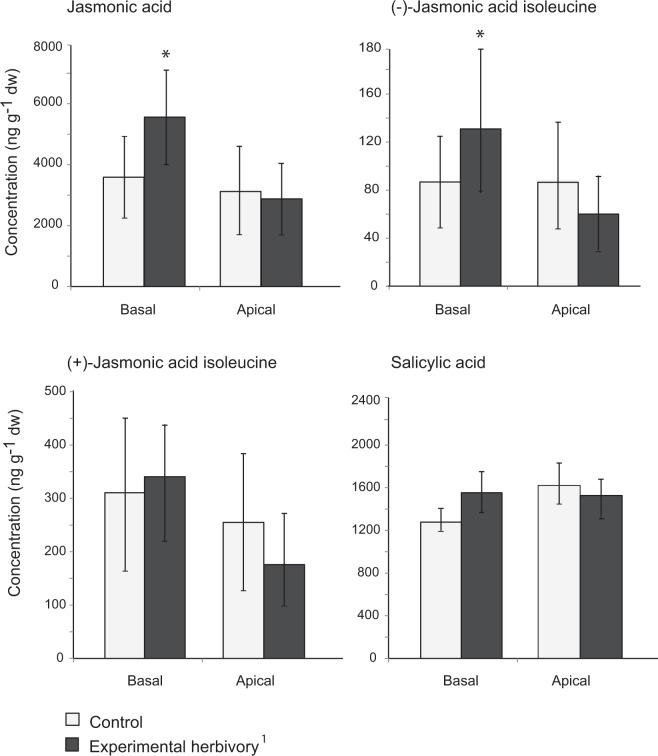
Effect of *L. dispar* herbivory on defense related phytohormones (salicylic acid, jasmonic acid, (+)-jasmonic acid isoleucine and (−)-jasmonic acid isoleucine) in the apical and basal portions of experimental herbivory vs. control branches. ^1^Experimental herbivory was only applied in the basal portion of the branch. Mean ± SE. N = 6. Asterisks (*) depict significant differences with a probability value (p-value) below 0.05 after a paired T-test.

We found significantly higher levels of jasmonic acid (JA) and its isoleucine conjugate JA Ile-(−) in the experimentally damaged leaves (basal) than in control leaves at the same position (paired T-test; t = 3937, d.f. = 5, p = 0.011 for JA, and t = 2.716, d.f. = 5, p = 0.042 for JA-Ile(−)). No significant differences were found for the apical leaf pools nor for the other phytohormones tested (Fig. 4). Data for the JA and its isoleucine conjugates was transformed using their natural logarithm (LN) to meet normality assumptions.

## Discussion

Our results show that experimental herbivory by leaf-chewing *L. dispar* significantly altered the volatile emission of mature *P. nigra* trees growing in a natural population. The emission of most classes of volatiles increased 4-6-fold from herbivore-damaged leaves and there were distinct compositional changes. Transcript levels of genes encoding enzymes involved in volatile biosynthesis also showed a significant up-regulation in damaged leaves, indicating that these volatiles are synthesized *de novo*. We also found higher levels of jasmonic acid (JA) and its (−)-JA-Ile conjugate in herbivore-damaged foliage. However, we found no evidence of a systemic induction in volatile emission, gene up-regulation, or increased phytohormone levels in the adjacent undamaged foliage.

This study shows that mature trees in the field have a robust response to herbivory, producing HIPVs even after previous natural herbivory and being exposed to naturally occurring environmental changes. A previous study on *P. nigra* saplings^36^ identified similar compounds as being important in distinguishing between the volatile blends of herbivore-damaged and undamaged leaves. 2- and 3-Methylbutyraldoximes, (*Z*)-3-hexenol, (*E*)-β-ocimene, and 4,8-dimethyl-1,3,7-nonatriene (DMNT) are characteristic of the herbivore-damaged foliage of both young and mature trees. Thus these compounds could have a role as “honest signals” in indirect defence by conveying specific information on herbivore location and abundance to natural enemies^42,43^. In support of this theory, 2-methylbutyraldoxime and (*Z*)-3-hexenol were previously identified as attractants of herbivore enemies under field conditions in the same floodplain forest studied here, and 2- and 3-methylbutyraldoximes, (*E*)-β-ocimene, DMNT and a (*Z*)-3-hexenol isomer, (*E*)-2 hexenol, were found to be physiologically active in a dose-dependent manner on the antennae of a *L. dispar* parasitoid (*Glyptapanteles liparidis*)^36^. Furthermore, aldoximes had a negative dose-dependent effect on larval survival^40^ and experienced *L. dispar* caterpillars avoided DMNT in laboratory trials, indicating that these compounds may also play a role in direct defence^44^.

We found significantly higher levels of JA and its (−)-JA-Ile conjugate at the local sites of experimental herbivory, but not in the controls or undamaged adjacent foliage. This is consistent with another field study on mature *P. nigra* of the same population, performed shortly after leaf flush, which also found a significant induction of JA and JA-Ile in herbivore-wounded leaves, but not of salicylic acid (SA)^45^. However, a laboratory study using *P. nigra* saplings obtained from branch cuttings from the same population, reported a significant induction of both JA and SA at the local sites upon herbivore damage^36^. These contrasting results suggest that signalling patterns can vary for plants of the same species (and possibly genotypes or even individuals) under different conditions. JA is an essential component of systemic signalling as evidenced by grafting studies using tomato mutants, which show that systemic signalling requires both the biosynthesis of jasmonic acid at the site of wounding and the ability to perceive a jasmonate signal in remote tissues, as well as the presence of other signalling molecules that amplify jasmonate production in vascular tissues^46^. In this sense, the absence of an induced systemic response is consistent with the lack of a significant increase in JA levels in the apical foliage of the experimental herbivory treatment.

Contrary to our results, previous studies investigating volatile responses to herbivory have frequently detected increased systemic emission (mainly terpenes) in foliage adjacent to the sites of damage. However, these studies were all performed using previously undamaged plants growing under controlled conditions. For instance, a seminal study by Turlings and Tumlinson^14^ reported the systemic emission of linalool, DMNT, and (3*E*,7*E*)-4,8,12-trimethyl1,3,7,11-tridecatetraene (TMTT) in corn seedlings under greenhouse conditions after *Spodoptera exigua* damage. Feeding by *Pieris brassicae* caterpillars on the lower leaves of Brussels sprouts (*Brassica oleracea* var. *gemmifera*) was also found to trigger volatile release from undamaged upper leaves in particular α-humulene and (*E*)-β-caryophyllene^18^. A study using potted saplings hybrid poplar (*P. trichocarpa x deltoides*) under greenhouse conditions found that *Malacosoma disstria* feeding induced local and systemic diurnal emissions of the terpenes (−)-germacrene D, (*E*)-β-ocimene, linalool, (*E*)-DMNT, and (*E,E*)-α-farnesene^13^. A similar pattern was also observed in our own previous study using *P. nigra* saplings^36^.

Upon closer inspection, some studies suggest that the induction of volatile emission from undamaged tissue requires different conditions in different plant species. For example, corn seedlings expressed a systemic response after only 5 to 6 h of herbivory^14^, while it took cotton plants about 48 h to do so^47^. The authors suggested that these differences could be related to the plants’ life history (annual vs. perennial) and the presence of constitutive chemical defences (i.e., well-defended plants will ‘wait’ longer to trigger systemic responses)^47^. Another study on Brussels sprouts (*Brassica oleraceae* var. *gemmifera*) – which are known to be chemically well defended – also suggested that there might be a threshold level of damage required to elicit systemic responses. In this case, systemic induction did not occur at low levels of infestation (5 early instar *Pieris brassicae*) even if caterpillars were feeding on the same leaf for a prolonged period of time (3 days)^18^.

At this stage, we are unable to determine whether the absence of a systemic response in this study is a general feature of mature poplar trees or it is due to lacking the appropriate conditions required for its induction. It is conceivable, that as plants mature, changes in size and function could influence resource allocation to plant defense^48,49^ based on changes in the costs and benefits of different defence mechanisms (e.g., direct vs. indirect or constitutive vs. induced), and that mature trees will allocate fewer resources to defence during and after the reproductive season, since there are high energy costs associated with reproduction in woody dioecious species like poplar^50,51^. However, other aspects, such as differences in vigour between upper and lower branches in the canopy, reduced light availability in the lower branches, location of the damage (apical vs. basal), and the history of herbivory and abiotic stress, could also influence the induction of defence responses. Further studies are needed to elucidate if mature Polar trees in general are able to induce a systemic defense response to herbivory, and if so, under which environmental conditions this happens. Also, sex-related differences in tree defense responses should be considered in future studies.

Taken together, these results emphasize the importance of both laboratory and field studies to obtain a full understanding of the spectrum of plant defence responses. Experiments performed under carefully controlled conditions allow researchers to determine the effect of a single herbivore or pathogen without the interference of others, and to elucidate the mechanisms of defence against a background of minimal environmental variation. However, experiments in the field permit much more realistic assessments of the inducibility of defences and provide information about the ecological significance of these responses.

## Methods

### Plant and insect material

#### Populus nigra

All experiments were performed in old growth *P. nigra* trees belonging to a natural black poplar population on Küstrin-Kietz island (52°34′1″N, 14°38′3″E, elevation: approx. 20 m above sea level) in the Oder River in northeastern Germany during the late spring of 2011. The population comprises around 350 trees of mainly one age class (~60 - 70 years old). Mean annual air temperature in the site is 11.5 °C, mean annual precipitation 402.3 mm.

#### Lymantria dispar

*L. dispar* caterpillars hatched from egg clutches (kindly provided by Hannah Nadel, of the Animal and Plant Health Inspection Service (APHIS) of the U.S. Department of Agriculture) and were reared on artificial gypsy moth diet (MP Biomedicals LLC, Illkirch, France) until two days before the experiments started. The caterpillars were then fed with leaves from *P. nigra* to get accustomed to this diet. We used fourth and fifth instar caterpillars for the experiment.

### Experimental set-up

Six branches, with fully expanded leaves, in the lower part of the canopy of nine old-growth *P. nigra* trees were selected for volatile collections. The branches were split into two sections, apical and basal **(**Fig. 1A**)**. Experimental herbivory was inflicted on the basal half of three branches per tree by confining seven caterpillars in this section; the adjacent apical section was left undamaged. The basal and apical sections of three branches without experimental herbivory were used as controls. The caterpillars were caged in nylon mesh cylinders and allowed to feed for around 40 h before the collection of volatiles and leaf material. The caterpillars were removed from the branches just before volatile measurement at day two.

### Plant harvest and herbivory measurement

Right after the volatile collections, the basal and apical leaf pool of each branch was harvested separately. The leaf blades were cut off and all leaves from each section were photographed.

Damaged leaf areas were reconstructed and calculated using Adobe Photoshop®. Five representative leaves from each pool were oven-dried at 80 °C, while the rest were immediately frozen in liquid nitrogen for gene expression and phytohormone analyses. Dry weights for the whole sample were extrapolated using the relationship between leaf area and dry weight. Thus volatile emission was calculated in nanograms per gram of dry weight per hour (ng g^−1^ (dw) h^−1^).

### Volatile collection and analysis

Volatiles were collected in the field from the apical and basal sections of control and experimental herbivory branches using a dynamic push-pull system. Foliage was enclosed in commercial polyethylene terephthalate (PET) bags (Toppits® Bratschlauch, Melitta, Minden, Germany), and volatiles were trapped for 2 h with 20 mg Super-Q filters as previously described^36^. Filters were eluted with 200 µl of a dichloromethane solution with 10 ng/µl of nonyl acetate as an internal standard.

Qualitative and quantitative analysis of volatiles was conducted using an Agilent 6890 Series gas chromatograph coupled to an Agilent 5973 quadrupole mass selective detector (interface temp.: 270 °C; quadrupole temp.: 150 °C, source temp.: 230 °C, electron energy: 70 eV) and a flame ionization detector operated at 300 °C. The constituents of the volatile bouquet were separated with a DB-5MS column (Agilent, Santa Clara, CA, USA, 30 m × 0.25 mm × 0.25 µm) and He (MS) or H_2_ (FID) as carrier gas. 1 µl of the sample was injected splitless at an initial oven temperature of 40 °C. The temperature was held for 2 min and then increased to 155 °C with a gradient of 7 °C min^−1^, followed by a further increase to 300 °C with 60 °C min^−1^ and a hold for 3 min.

Compounds were identified by comparison of retention times and mass spectra to those of authentic standards obtained from Fluka (Seelze, Germany), Roth (Karlsruhe, Germany), Sigma (St, Louis, MO, USA) or Bedoukian (Danbury, CT, USA), or to reference spectra in the Wiley and National Institute of Standards and Technology libraries and in the literature (Joulain and König, 1998)^52^. Some compounds not commercially available were kindly provided by Wilfried A. König (Hamburg) as characterized essential oil samples of *Oreodaphne porosa* and *Aloysia sellowii*. The absolute amounts of all compounds were determined based on their FID peak area in relation to the area of the internal standard using the effective carbon number (ECN) concept as described by Scanion and Willis^53^. Samples from branches within the same tree and treatment were pooled, averaged and used as a single value for that individual tree for further statistical analyses.

### Isolation and characterization of *PnTPS3*

The N-terminal truncated open reading frame of *PnTPS3* lacking the first 57 nucleotides was amplified from cDNA made from herbivore-damaged leaves of *P. nigra* genotype 2 using the primers listed in Table S2. The gene was inserted as a *Bsa*I fragment into the expression vector pASK-IBA7-plus (IBA-GmbH, Göttingen, Germany) and fully sequenced. This terpene synthase construct was expressed in *Escherichia coli* and the resulting protein purified following the procedure described in Danner *et al*.^38^. To determine the catalytic activity of PnTPS3, enzyme assays containing 50 μl of the bacterial extract and 50 µl assay buffer (10 mM Tris-HCl, pH 7.0, 1 mM dithiothreitol, 10% (v/v) glycerol) with 10 μM GDP) and 10 mM MgCl_2_ were performed in a Teflon-sealed, screw-capped 1 ml GC glass vial. An SPME (solid phase microextraction) fibre consisting of 100 µm polydimethylsiloxane (Supelco, Bellefonte, PA, USA) was placed in the headspace of the vial that was incubated at 30 °C for 1 h. For analysis of the adsorbed reaction products, the SPME fibre was directly inserted into the injector of the gas chromatograph. The TPS enzyme products were analysed and identified using GC-MS as described above for poplar volatiles (Fig S1). The GC was operated with a DB-5MS column (Agilent, Santa Clara, USA, 30 m × 0.25 mm × 0.25 µm). The sample (SPME) was injected without split at an initial oven temperature of 50 °C. The temperature was held for 2 min, then increased to 240 °C with a gradient of 7 °C min^−1^, and further increased to 300 °C with a gradient of 60 °C min^−1^ and a hold of 2 min. The enzyme product (*E*)-β-ocimene was identified using an authentic standard obtained from Sigma (St. Louis, MO, USA).

### qRT-PCR of volatile biosynthesis genes

For gene transcript abundance, we used leaf material from the basal portions of control and experimental herbivory leaves of six trees. RNA was isolated from frozen material and cDNA was generated following the method described in Maffei *et al.* 2011^12^. Gene-specific primers for *PnTPS3* and *PnCYP79D6-v4* were designed to give a predicted melting temperature of about 60 °C, a primer length in the range of 20–25 nt and an amplicon length between 100–200 bp.

Primer specificity was confirmed by agarose gel electrophoresis, melting curve analysis, standard curve analysis and sequence verification of cloned PCR amplicons^36^. Specific primers for *ubiquitin* were used as reference genes^54^. The following PCR conditions were applied for all reactions: Initial incubation at 95 °C for 3 min followed by 40 cycles of amplification (95 °C for 20 s, 60 °C for 20 s). Reactions were measured during the annealing and the extension step of each cycle. Data for the melting curves were gathered at the end of the 40 cycles from 55 °C to 95 °C. Samples were run in triplicates using Brilliant^®^ III SYBR^®^ Green QPCR Master Mix (Stratagene, Agilent) with ROX as reference dye. Reactions were set up according to manufacturer’s instructions. Each cDNA was diluted 1:3 and 1 µl of the diluted cDNA was used in qPCR reactions. All samples were run on the same PCR machine (MxPro – Mx3000P, Stratagene, Agilent) in an optical 96-well plate.

### Phytohormone analysis

For phytohormone analysis, we used leaf material from the basal and apical portions of control and experimental herbivory branches of six trees. Starting from frozen material, a subsample of 20 mg of finely ground lyophilized leaf material was extracted with methanol containing 40 ng of 9, 10-D_2_-9,10-dihydrojasmonic acid, D_4_-salicylic acid (Sigma-Aldrich), and 8 ng of jasmonic acid-^13^C_6_-isoleucine conjugate as internal standards. The extracts were analysed on an Agilent 1200 HPLC system (Agilent Technologies) coupled to an API 3200 tandem mass spectrometer (Applied Biosystems) equipped with a Turbospray ion source as previously described^45^.

Separation was achieved on a Zorbax Eclipse XDB-C18 column (50 × 4.6 mm, 1.8 µm, Agilent) with a gradient of formic acid (0.05%) in water and acetonitrile. The mass spectrometer was operated in negative ionization mode with multiple reaction monitoring (MRM). Analyst 1.5 software (Applied Biosystems) was used for data acquisition and processing. Phytohormones were quantified relative to the signal of their corresponding internal standard.

### Statistical analysis

All statistical analyses were performed using the open source software R Studio (http://www.r-project.org/; Crawley 2007) and SPSS for Windows (SPSS, Chicago, IL, USA). When standard statistical assumptions such as normal distribution were not met, data were transformed using the natural logarithm (ln).

A paired T-test was used to estimate the changes in leaf area loss between the *L. dispar* damaged leaf pool and the basal control, as well as between the apical and basal portions of the *L. dispar*-damaged branch.

For volatile emission paired T-tests were performed to test for differences in volatile emission between the treatments (experimental herbivory and control) for both the apical and basal sections of the branches. To classify the composition of volatiles emitted from *P. nigra* in damaged and adjacent foliage of herbivore infested and control trees, and to assign individual volatile compounds (variables) to these experimentally defined groups, we used the machine learning algorithm “random forest”^55^, a multivariate statistical tool. Ntree = 100,000 bootstrap samples were drawn with mtry = 7 variables (volatiles) randomly selected at each node. The importance of each variable for the classification is expressed as the mean decrease in accuracy (MDA). Furthermore, “random forest” returns an out of bag (OOB) error rate for each classification.

Paired T-tests were used to evaluate differences between expression of the volatile biosynthetic genes *PnTPS3* and *PnCYP79D6-v4* in the basal leaf pools of experimental-herbivory and control branches (n = 6), as well as for the emission of their biosynthetic products, (*E*)-β-ocimene and the methybutyraldoximes. We calculated the Pearson correlation coefficient (R^2^) to establish the relation between the emission of these compounds and the amount of herbivory and its significance.

Paired T-tests were used to estimate the changes in the concentration of poplar defence hormones between the control treatment and the experimental herbivory treatment of the apical and basal leaf pools separately.

## Supplementary information

Supplemental Information

## Data Availability

The data will be made available upon request.
