# Supplementary material for: Herbivore-induced volatile emission from old-growth black poplar trees under field conditions
Source: Sci Rep. 2019 May 22;9:7714. doi: 10.1038/s41598-019-43931-y (PMC6531464; doi:10.1038/s41598-019-43931-y)
Supplement: Supplementary file 1 — Supplemental Information [file 41598_2019_43931_MOESM1_ESM.pdf]

Supplemental Information

Herbivore-induced volatile emission from old-growth black poplar trees under field conditions

Andrea Clavijo McCormick[1,2], Sandra Irmisch[1,3], G. Andreas Boeckler[1], Jonathan Gershenzon[1], Tobias G. Köllner[1] & Sybille B. Unsicker[1]*

[1]Max Planck Institute for Chemical Ecology, Department of Biochemistry, Hans-Knöll-Straße 8, 07745 Jena, Germany

[2] Current address: Massey University, College of Sciences, Tennent Drive, 4410 Palmerston North, New Zealand

[3] Current address: Michael Smith Laboratories, University of British Columbia, 2185 East Mall, Vancouver, BC, Canada V6T 1Z4

*Corresponding author: Sybille B. Unsicker, Max Planck Institute for Chemical Ecology, Hans-Knöll-Straße 8, 07745 Jena, Germany, Phone: +49 3641 571328, Fax: +49 3641571302, Email: sunsicker@ice.mpg.de

Table S1. Volatile compounds of old-growth *Populus nigra* released from control leaves and leaves after experimental herbivory by gypsy moth (*Lymantria dispar*) for 40 h. Emission rates are displayed as means ± SEM in ng g^{-1} dw h^{-1}. The compounds were either identified by authentic standards (STD), mass spectra (MS) or by comparison to compounds of the essential oils of *Alloysia sellowi* (Alloysia) and *Oreodaphne porosa* (Phoebe) which were kindly provided by W.A. König (Hamburg). ST = Sesquiterpene. Each replicate corresponds to the average of three branches per treatment per tree (n=9).

	Control				Experimental herbivory[1]			
	Basal		Apical[2]		Basal		Apical	
	MEAN	± SEM	MEAN	± SEM	MEAN	± SEM	MEAN	± SEM
***GLV*[3]**								
(*Z*)-3-Hexenol	2.050	0.894	3.514	1.039	23.459	11.629	9.536	5.289
(*Z*)-3-Hexenylacetate	14.040	8.843	12.626	4.423	31.146	10.268	22.271	8.549
(*Z*)-3-Hexenyl isobutyrate	0.371	0.270	0.733	0.343	1.006	0.456	1.043	0.651
(*Z*)-3-Hexenyl isovalerate	3.084	1.492	3.901	1.298	12.237	3.698	5.646	1.753
Monoterpenes								
α-Pinene	1.200	0.240	2.668	0.855	2.064	0.639	2.365	0.436
Camphene	11.104	1.049	9.672	0.847	11.285	1.830	9.168	0.914
Sabinene	0.726	0.244	2.021	0.689	2.627	1.092	1.256	0.325
Myrcene	3.985	0.992	14.194	10.017	7.446	2.033	7.968	2.522

Limonene	8.448	3.869	6.836	2.347	7.559	2.348	8.255	3.376
1,8-Cineole	1.364	0.463	4.835	2.329	4.823	1.938	2.524	0.756
(*Z*)-β-Ocimene	6.495	2.174	11.706	5.784	22.067	4.250	9.052	1.710
(*E*)-β-Ocimene	42.969	17.093	47.189	8.229	227.542	86.338	57.924	16.438
(*Z*)-Linalool oxide	4.003	1.263	8.162	5.322	14.873	5.304	4.911	0.923
Linalool	13.600	8.974	36.939	30.533	96.735	49.420	14.110	6.152
Camphor	0.154	0.104	0.510	0.263	1.022	0.434	0.551	0.357
Borneol	0.174	0.116	0.942	0.637	1.575	0.555	0.116	0.082
α-Terpineol	0.408	0.185	1.321	0.774	1.378	0.655	0.478	0.196
Sesquiterpenes								
α-Cubebene	0.703	0.314	0.980	0.428	3.105	0.802	0.655	0.239
α-Copaene	2.039	0.588	2.602	0.831	6.494	0.726	2.534	0.503
β-Bourbonene	1.798	0.749	2.688	1.341	5.318	1.721	2.219	0.744
β-Cubebene	1.868	0.756	2.242	1.074	8.148	1.733	1.901	0.672
(*E*)-β-Caryophyllene	13.692	4.267	18.059	5.100	41.295	7.407	17.035	2.740
α-Humulene	3.057	0.868	5.862	2.165	12.099	2.880	5.055	1.036
Unidentified ST1	0.637	0.280	1.321	0.571	2.072	0.617	0.683	0.206
Germacrene-D	10.256	3.641	15.554	7.805	52.795	15.864	11.278	3.625
Unidentified ST2	3.278	1.651	4.462	1.984	18.274	3.443	3.279	0.993

Unidentified ST3	0.310	0.181	2.195	1.230	2.456	0.793	0.461	0.200
Unidentified ST4	0.539	0.304	0.858	0.554	1.633	0.642	0.530	0.188
(*E*,*E*)-α-Farnesene	15.577	7.912	15.274	6.389	105.792	36.210	11.832	3.202
γ-Cadinene	1.335	0.572	2.611	1.280	3.218	0.895	1.267	0.397
δ-Cadinene	2.572	1.048	4.737	1.955	8.022	2.358	2.430	0.788
Nerolidol	12.333	12.001	7.155	6.297	13.377	10.383	5.465	3.918
Homoterpenes								
(*Z*)-DMNT	2.792	1.204	2.781	0.962	16.891	4.502	2.390	0.613
(*E*)-DMNT	145.140	86.664	102.289	50.037	774.039	293.893	88.528	32.159
Aromatics								
Benzaldehyde	5.540	1.069	4.663	0.672	7.173	1.999	6.048	1.039
Benzyl alcohol	1.899	0.934	0.724	0.417	0.286	0.218	1.446	0.393
Salicyl aldehyde	1.928	0.677	6.241	4.325	3.577	1.923	1.551	0.552
Methyl salicylate	1.936	1.513	1.531	0.697	3.178	1.428	1.476	0.654
Eugenol	0.233	0.121	1.362	0.450	4.780	2.220	0.372	0.227
(*Z*)-Jasmone	1.176	0.380	2.397	1.139	3.881	1.110	2.174	0.362
Nitrogenous compounds								
(Z)-2-Methylbutyraldoxime	0.497	0.249	2.252	1.454	14.206	4.460	1.121	0.540
(Z)-3-Methylbutyraldoxime	1.783	0.497	2.434	1.024	11.511	3.524	2.152	0.546
(*E*)-2- + 3-Methylbutyraldoxime	4.366	1.566	7.559	4.047	57.315	15.572	5.158	1.151

Benzylcyanide	3.464	1.369	6.354	3.127	19.637	6.040	2.989	1.284
Indole	0.030	0.030	0.000	0.000	1.793	0.679	0.000	0.000
2-Phenylnitroethane	0.028	0.028	0.150	0.150	1.315	0.892	0.000	0.000
Others								
Isoamylacetate	6.173	0.591	5.273	0.502	10.094	2.583	4.964	0.476
3-Methyl-2-butenal	11.323	0.998	9.720	0.842	11.663	1.979	9.655	1.093
3,7-Dimethyldecane	1.539	1.018	10.601	8.563	4.933	2.503	4.536	2.085
4-Methyldecane	0.800	0.516	5.374	4.055	2.693	1.398	2.176	1.054
Decanal	8.146	1.362	8.548	2.225	11.397	1.979	11.943	2.562
Nonanal	15.045	2.462	18.179	4.277	19.094	3.963	21.018	3.546
Heptadecane	1.663	0.831	4.677	3.305	2.580	1.248	2.252	0.819
Tetradecane	3.937	1.125	8.394	4.895	6.907	2.166	5.678	1.128
Pentadecane	2.106	1.293	12.063	8.491	7.696	3.224	5.108	1.601

[1] Experimental herbivory was only inflicted in the basal portion of the experimental herbivory branches.
[2] The apical control treatment has only eight replicates due to the loss of one sample.
[3] Saturated and unsaturated six-carbon aldehydes, alcohols and their esters

Table S2: List of oligonucleotides used in this study. qPCR, primer were used for qRT-PCR analysis; cloning, primer were used for the amplification and cloning of *PnTPS3*.

name	sequence	usage
PnTPS3-fwd	ATGCCGAGGCATCCTCTGCC	cloning
PnTPS3-rev	TTAATGAAATGAAATGGGTTCAATTA	cloning
Ubi_fwd	GTTGATTTTTGCTGGGAAGC	qPCR
Ubi_rev	GATCTTGGCCTTCACGTTGT	qPCR
PnTPS3-qRT-fwd	CGATCGGTATCAGGAACTGTC	qPCR
PnTPS3-qRT-rev	GCTATCTCTGCTGATGAAGTG	qPCR
PnCYP79D6v4-fwd	GAGAGACTTGTCCAAGAATCAG	qPCR
PnCYP79D6v4-rev	GAAGTAGTTGGCAACTGTTGT	qPCR
